# HMGB1 contributes to SASH1 methylation to attenuate astrocyte adhesion

**DOI:** 10.1038/s41419-019-1645-7

**Published:** 2019-05-28

**Authors:** Ronghua Wu, Yingying Yan, Chao Ma, Han Chen, Zhangji Dong, Yongjun Wang, Yan Liu, Mei Liu, Liu Yang

**Affiliations:** 1 Key Laboratory of Neuroregeneration of Jiangsu and Ministry of Education, Co-innovation Center of Neuroregeneration, Nantong University, Nantong, China; 2 Department of Neurosurgery, Affiliated Hospital of Nantong University, Nantong, China

**Keywords:** Cell adhesion, Tumour-suppressor proteins

## Abstract

SAM and SH3 domain-containing 1 (SASH1), a scaffold protein, is regarded as a tumor suppressor. Recent studies have verified the decreased expression of SASH1 in many tumors. Our previous clinical investigation found that SASH1 was widely expressed in normal brain tissues but reduced or absent in glioma tissues. However, the functions of SASH1 in normal astrocytes and the reasons for the reductions in SASH1 levels in glioma tissues are unclear. In this study, we found that in astrocytes, SASH1 functions in cell adhesion. We observed that knockdown of SASH1 expression in cultured astrocytes significantly decreased cell adhesion and increased invasion. Conversely, overexpression of SASH1 in C6 cells markedly promoted cell adhesion and decreased cell invasion. In addition, we found that the expression level of one member of the integrin family, integrin β8, was significantly reduced in SASH1-downregulated astrocytes and elevated in SASH1-upregulated C6 cells. Furthermore, the results of methylation and ChIP assays showed that the methylation level of the SASH1 gene was markedly higher in C6 cells than in astrocytes and that HMGB1 could bind to the CpG islands of the SASH1 gene. HMGB1 overexpression in astrocytes significantly increased the methylation level of the SASH1 gene. This study reveals, for the first time, that HMGB1 contributes to the methylation of the SASH1 gene, and our findings suggest that methylation downregulates the expression of the SASH1 gene and later reduces integrin β8 expression, thereby reducing cell adhesion and promoting cell migration.

## Introduction

The expression of SAM and SH3 domain-containing 1 (SASH1) was first reported to be significantly decreased in breast cancer samples by Zeller in 2003^1^. The SASH1 gene is widely expressed in normal human tissue. This gene regulates cell growth, proliferation, and apoptosis and is involved in the development of a variety of diseases. Current studies regard SASH1 as a tumor suppressor gene. SASH1 gene function is reduced or absent in most human tumor cells, such as lung cancer^2^, gastric cancer^3^, colon cancer^4,5^, cervical cancer^6^, ovarian carcinoma^7^, and thyroid cancer cells^8^. Our previous studies found that SASH1 expression in high-grade gliomas was significantly lower than that in low-grade gliomas and that low SASH1 expression was also correlated with poor prognosis^9^. When the SASH1 gene is overexpressed in glioma cells, cell invasion, and cell proliferation decrease^10^. However, the mechanism by which SASH1 influences these biological behaviors in normal glia is unclear, and the specific factor that downregulates SASH1 expression has not been thoroughly elucidated to date.

The annual incidence rates of most human tumors have declined; however, the incidence of brain glioma is still increasing, revealing that it is one of the least curable types of clinical tumors^11^. High-grade gliomas usually grow invasively, showing no clear boundaries with surrounding normal tissue. Uncovering the possible mechanism of glioma invasion will be of benefit to clinical therapeutics. As a scaffold protein, SASH1 has been reported to play an important role in the regulation of signal transduction. SASH1, together with related molecules, regulates cytoskeletal proteins and promotes cell and matrix adhesion^12^. In addition, Zhou et al. found that SASH1 affects E-cadherin signaling to regulate transepithelial migration^13^.

Therefore, in this study, we manipulated SASH1 gene expression using siRNA in cultured astrocytes and compared these cells to C6 glioma cells transfected with Adv4-SASH1. We further investigated changes in relevant biological characteristics of the cells and the effects of SASH1 on astrocyte adhesion.

## Results

### SASH1 expression levels are related to cell proliferation

**Fig. 1:**
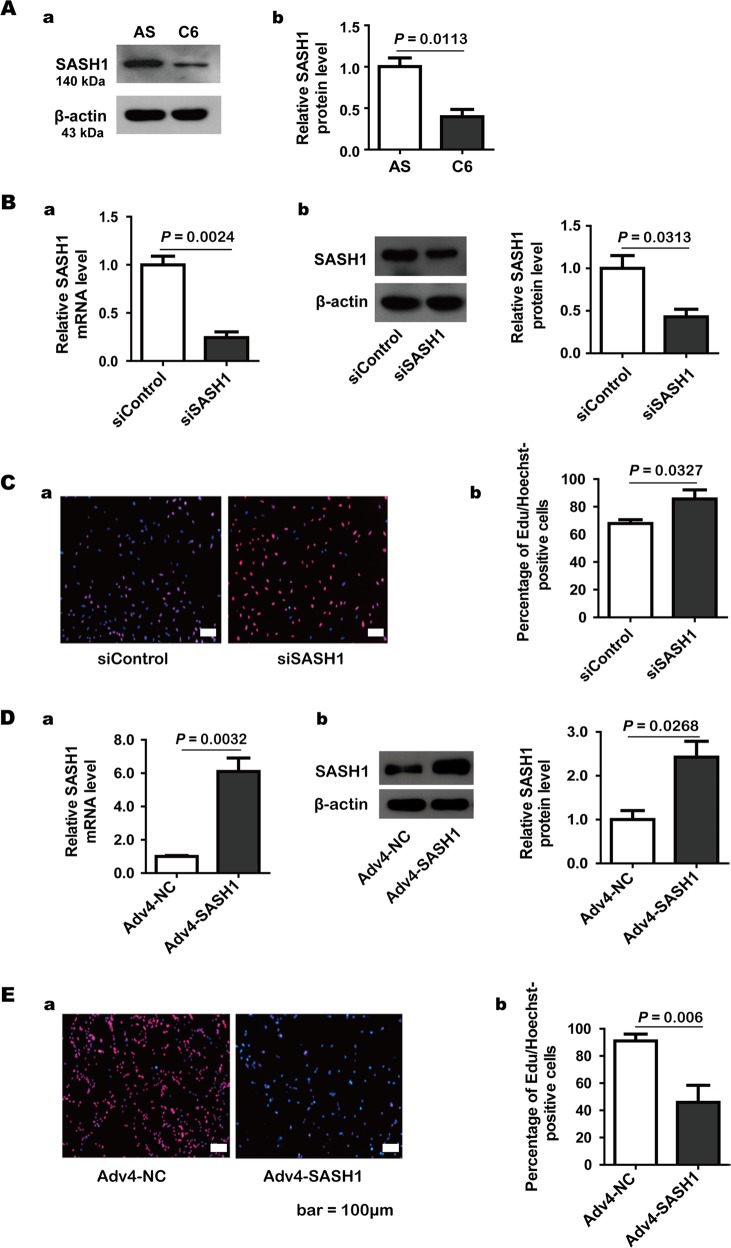
Original and manipulated expression of SASH1 in astrocytes and C6 glioma cells. **A** The original SASH1 protein expression in C6 rat glioma cells and cultured rat astrocytes. β-Actin was used as the internal control. a Representative Western blots. b The statistical results. Astrocytes vs. C6 cells, *n* = 3, *P* = 0.0113. **B** The mRNA and protein levels of SASH1 in astrocytes after treatment with SASH1 siRNA. a The statistical results of qRT-PCR analysis. b Representative Western blots (left) and the statistical results (right). SASH1 siRNA (siSASH1) vs. control siRNA (siControl), *n* = 3, *P* = 0.0024 (mRNA), *P* = 0.0313 (protein). **C** The results of the EdU assay on cultured astrocytes. a Representative images of the EdU assay results in astrocytes after siControl and siSASH1 treatment. Panel b The statistical results. siSASH1 vs. siControl, *n* = 4, *P* = 0.0327. **D** The mRNA and protein levels of SASH1 in C6 cells after Adv4-SASH1 application. a The statistical results of qRT-PCR analysis. b Representative Western blots (left) and the statistical results (right). Adv4-SASH1 vs. control Adv4 vector (Adv4-NC), *n* = 3, *P* = 0.0032 (mRNA), *P* = 0.0268 (protein). **E** The results of the EdU assay on C6 glioma cells. a Representative images of the EdU assay results in C6 cells after Adv4-NC and Adv4-SASH1 treatment. b The statistical results. Adv4-SASH1 vs. Adv4-NC, *n* = 4, *P* = 0.006

First, we identified SASH1 expression using Western blotting, and the results are shown in Fig. 1A. SASH1 protein expression was almost 2.5-fold higher in astrocytes than in C6 glioma cells. After the astrocytes were treated with SASH1 siRNA for 3 days, the SASH1 mRNA and protein levels decreased to 25.6% and 42.9%, respectively, of the levels in the control siRNA group (Fig. 1B). In addition, we detected the effect of SASH1 siRNA on astrocyte proliferation using the EdU incorporation method, and the results, shown in Fig. 1C, showed that the proliferation ratio increased from 67.9% for the control siRNA-treated astrocytes to 85.6% for the SASH1 siRNA-treated astrocytes.

We applied AdV-SASH1 to C6 cells for 3 days and found that treatment with AdV-SASH1 increased the SASH1 mRNA and protein levels to 610.5% and 242.4%, respectively, of the control levels (in cells treated with control adenovirus) (Fig. 1D). We also detected the effect of SASH1 overexpression on C6 cell proliferation, and the results, as shown in Fig. 1E, indicated that the proliferation ratio decreased from 91.0% in the control cells to 45.9% in the Adv4-SASH1-overexpressing C6 cells.

These results showed that SASH1 expression is low in C6 glioma cells and high in cultured astrocytes. The SASH1 expression level could be effectively manipulated in C6 glioma cells or astrocytes by Adv4-SASH1 application or SASH1 siRNA treatment, respectively. In addition, we confirmed that the cell proliferation ratio is closely related to the SASH1 expression level.

### RNAseq analysis reveals that SASH1 protein depletion affects cell adhesion

**Fig. 2:**
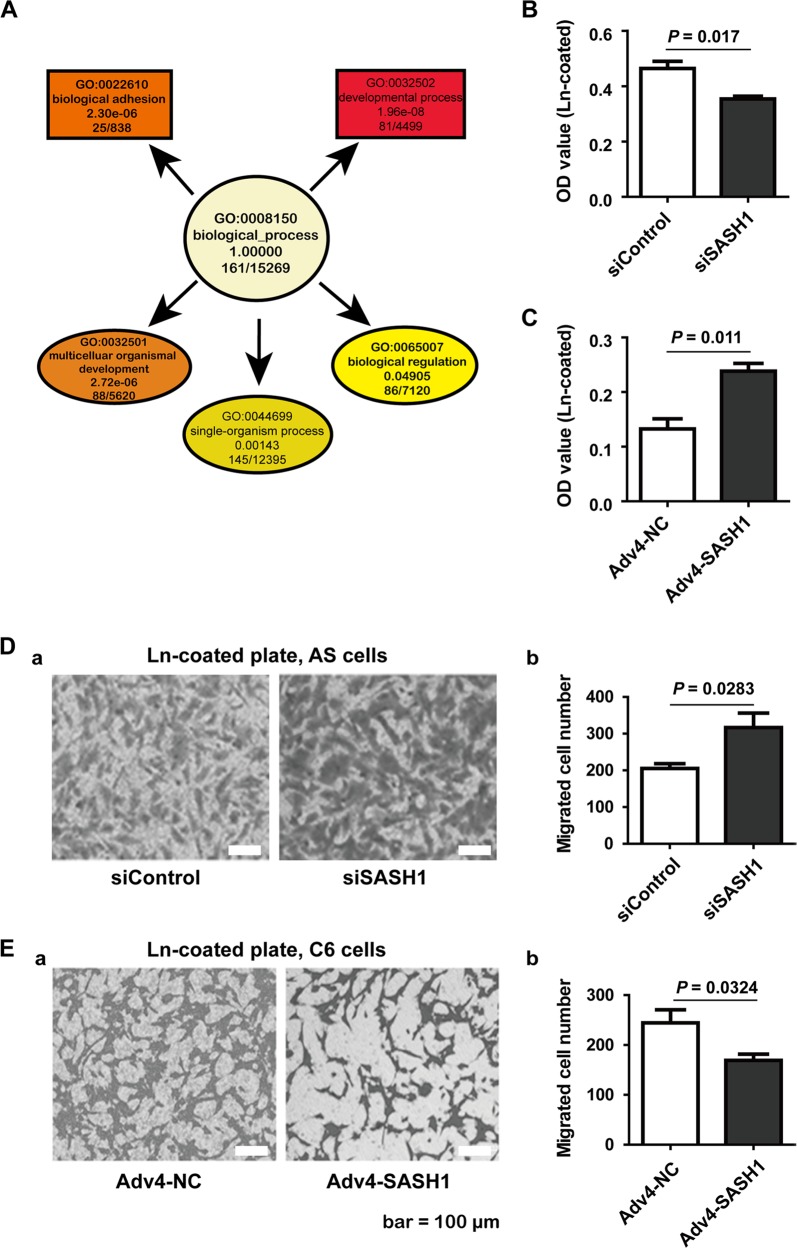
Effect of SASH1 on cell adhesion. **A** The results of GO analysis of the differential gene expression due to depletion of SASH1 were clustered in the biological adhesion and other biological processes categories. **B** The results of the cell adhesion assay showed that depletion of SASH1 expression attenuated cell adhesion when astrocytes were seeded on Laminin (Ln)-coated wells for 20 min. siSASH1 vs. siControl, *n* = 3, *P* = 0.017. **C** Overexpression of SASH1 in C6 cells significantly increased cell adhesion when C6 cells were seeded on Ln-coated wells for 20 min. Adv4-SASH1 vs. Adv4-NC, *n* = 3, *P* = 0.011. **D** The results of the cell invasion assay showed that depletion of SASH1 expression markedly increased cell invasion when astrocytes were seeded on Ln-coated upper chambers. a Representative images of the transwell assay results. b The statistical results. siSASH1 vs. siControl, *n* = 4, *P* = 0.0283. **E** Overexpression of SASH1 in C6 cells significantly decreased the ability of C6 cells to invade the Ln-coated upper chambers. a Representative images of the transwell assay results. b The statistical results. Adv4-SASH1 vs. Adv4-NC, *n* = 4, *P* = 0.0324

We used RNAseq analysis to investigate the effects of SASH1 depletion on cell function. The information on the differentially expressed genes is shown in the supplemental file named BiologicalInfoAnalysisReport. GO analysis revealed that SASH1 depletion significantly affected adhesion and other biological processes (Fig. 2A). Usually, cell adhesion is related to actin cytoskeletal reorganization^14^. The extracellular signal for actin polymerization is mostly dependent on Laminin^15^. Laminin is a major extracellular matrix (ECM) protein in the brain^16^. Therefore, we used a cell adhesion assay to detect whether Laminin could be involved in the effects on adhesion mediated by SASH1 protein expression.

After the astrocytes were transfected with control or SASH1 siRNA or the C6 cells were transfected with Adv4-NC or Adv4-SASH1, the cells were reseeded in Laminin-coated wells. As shown in Fig. 2B, C, the results of the cell adhesion assay showed that a loss of SASH1 function significantly decreased cell adhesion to the Laminin coating in 20 min (Fig. 2B), while a gain of SASH1 gene function markedly increased cell adhesion to the Laminin coating in 20 min (Fig. 2C).

We used a Transwell assay to detect whether cell invasion was affected by SASH1 protein expression. After astrocytes or C6 cells were subjected to SASH1 gene downregulation or upregulation, the cells were reseeded in the upper chambers, which were precoated with Laminin. After cell invasion for 16–18 h, the cells in the upper chambers were removed, and the bottom membranes were stained using crystal violet. The results showed that compared to control siRNA-treated astrocytes, SASH1 siRNA-treated astrocytes showed strong invasion on the laminin-coated membranes. In addition, compared to Adv4-NC application, Adv4-SASH1-mediated overexpression resulted in less invasion of C6 cells on the Laminin-coated membranes. These results revealed that the SASH1 expression level impacted cell invasion ability.

### The effect of SASH1 on cell invasion is related to integrin β8 expression

**Fig. 3:**
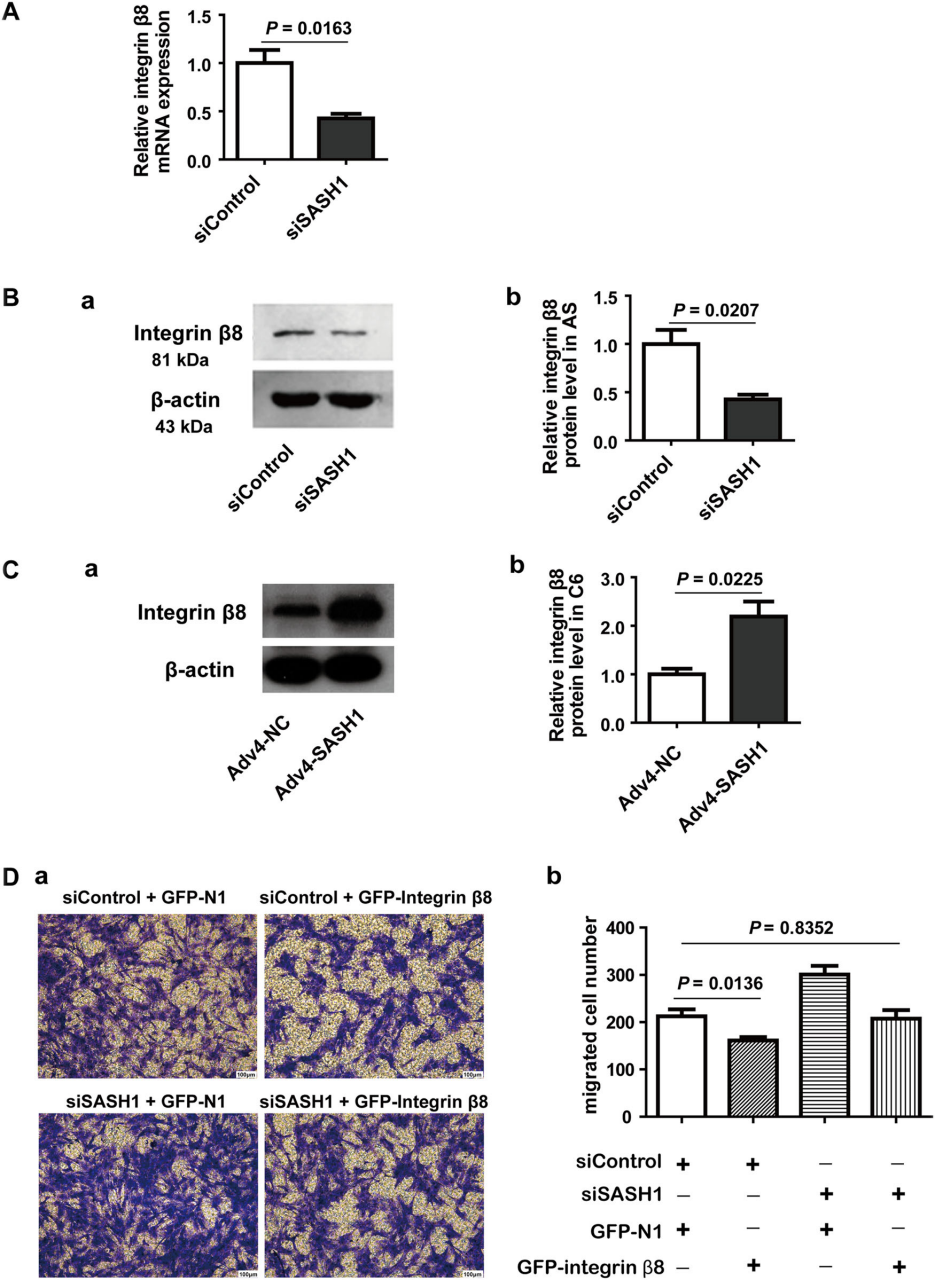
Effects of SASH1 on cell invasion and integrin β8 expression. **A** qRT-PCR results showing that the depletion of SASH1 expression by siSASH1 treatment could significantly decrease integrin β8 mRNA levels in astrocytes. *n* = 3, *P* = 0.0163. **B** The results of Western blot analysis showing that the expression of integrin β8 was significantly reduced in astrocytes after siSASH1 treatment. a Representative Western blots. b The statistical results. *n* = 3, *P* = 0.0207. **C** Integrin β8 protein levels were markedly increased in C6 cells after application of Adv4-SASH1. c Representative Western blots. d The statistical results. *n* = 3, *P* = 0.0225. **D** The results of the cell invasion assay showing that Integrin β8 overexpression plasmids attenuated the increases in cell invasion due to siSASH1 treatment. Astrocytes were transfected by electroporation with a combination of siRNA and plasmid. After culture for 3 days, trypsin-isolated cells were seeded on the Ln-coated upper chambers of Transwell plates. a Representative figures of the transwell assay results. b The statistical results. *n* = 4, actual *P*-values indicated in bar graphs

Next, we investigated which member of the integrin family was involved in the observed effects on cell invasion. We used a qRT-PCR method to detect alterations in integrin β1, β5, and β8 mRNA after SASH1 expression was changed. We found that integrin β8 mRNA expression was markedly decreased in SASH1-depleted astrocytes (Fig. 3A). Western blotting showed that integrin β8 protein expression was also decreased by 57% (Fig. 3B) in the SASH1-depleted astrocytes and that the expression of integrin β8 was significantly increased to 119% of control levels (Fig. 3C) in the C6 cells transfected with Adv4-SASH1. These results suggested that depletion of SASH1 resulted in the downregulation of integrin β8 expression.

Then, we detected the effect of integrin β8 overexpression on cell migration using the above Transwell method. As shown in Fig. 3D, integrin β8 overexpression significantly decreased cell migration (*P* = 0.0136), and integrin β8 attenuated the increased cell migration caused by SASH1 siRNA treatment (*P* = 0.8352).

### High methylation contributes to the decreased SASH1 expression in C6 glioma cells

We used methylation-specific PCR (MSP) and bisulfite genomic sequencing assays to examine the methylation statuses of the CpG islands of the SASH1 gene in astrocytes and C6 cells, referring to the methods in a previous study^17^.

**Fig. 4:**
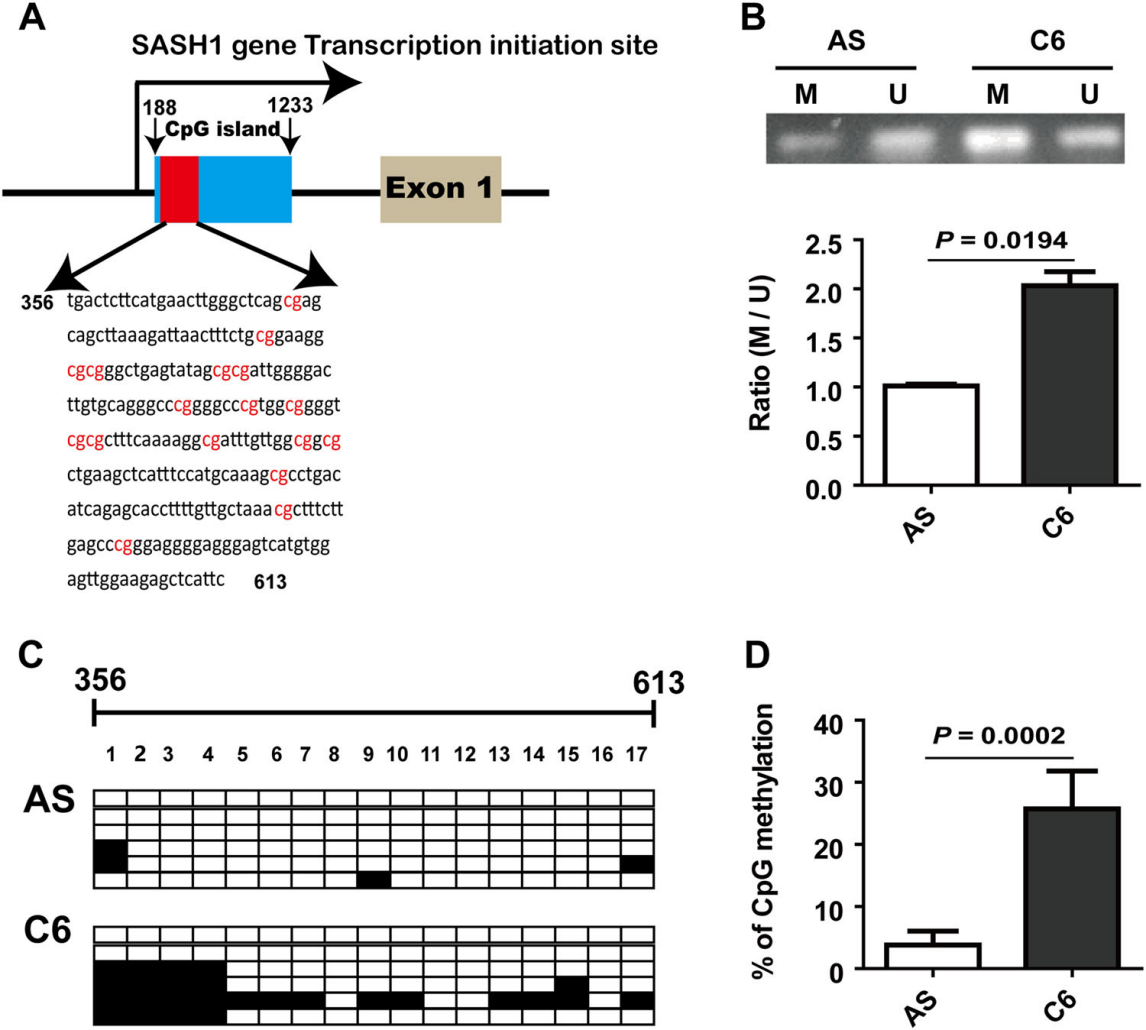
High methylation levels of the SASH1 gene in C6 glioma cells. **A** Schematic of a CpG island showing the locations of the 17 CpG sites (shown in red text) in the 5′ UTR of the SASH1 gene promoter. **B** Representative PCR results for the SASH1 promoter region using primers specific for methylated (M) and unmethylated (U) gDNA in astrocytes (AS) and C6 cells. The bar graph (lower panel) shows the M-to-U product ratios. C6 vs. AS, *n* = 3, *P* = 0.0194. **C** Each square indicates a clone of AS or C6 cells. Six clones were subjected to bisulfite sequencing. The clones methylated on individual CpG sites are labeled in black. **D** A significant increase in the percentage of methylated CpG sites was observed in C6 cells compared to AS cells. *P* = 0.0002

As shown in Fig. 4A, the predicted CpG islands exist in the 5′ UTR region (+188 bp to +1233 bp) of the rat SASH1 gene promoter. The representative figure shows methylated and unmethylated bands from AS and C6 cells. The ratio of M (methylated) CpG sites to U (unmethylated) CpG sites of the SASH1 gene was significantly increased in C6 cells compared to astrocytes (Fig. 4B). The results of DNA sequencing of the 258 bp PCR fragment (+356 bp to +613 bp) were obtained after the gDNA samples were treated with sodium bisulfite. The fragments (shown in Fig. 4A) containing 17 CpG sites, indicated with red text, were sequenced (Fig. 4C). The results revealed that compared with that in astrocytes, the degree of methylation of CpG sites within the chosen 258 bp fragment was significantly increased in C6 glioma cells (Fig. 4D), which was consistent with the results of the MSP assay. The hypermethylation of the SASH1 gene in C6 cells’ genomic DNA indicates that the transcription of SASH1, a tumor suppressor gene, is reduced. Therefore, SASH1 gene promoter methylation contributes to the downregulated SASH1 expression in glioma.

### HMGB1 contributes to the methylation of the SASH1 gene

**Fig. 5:**
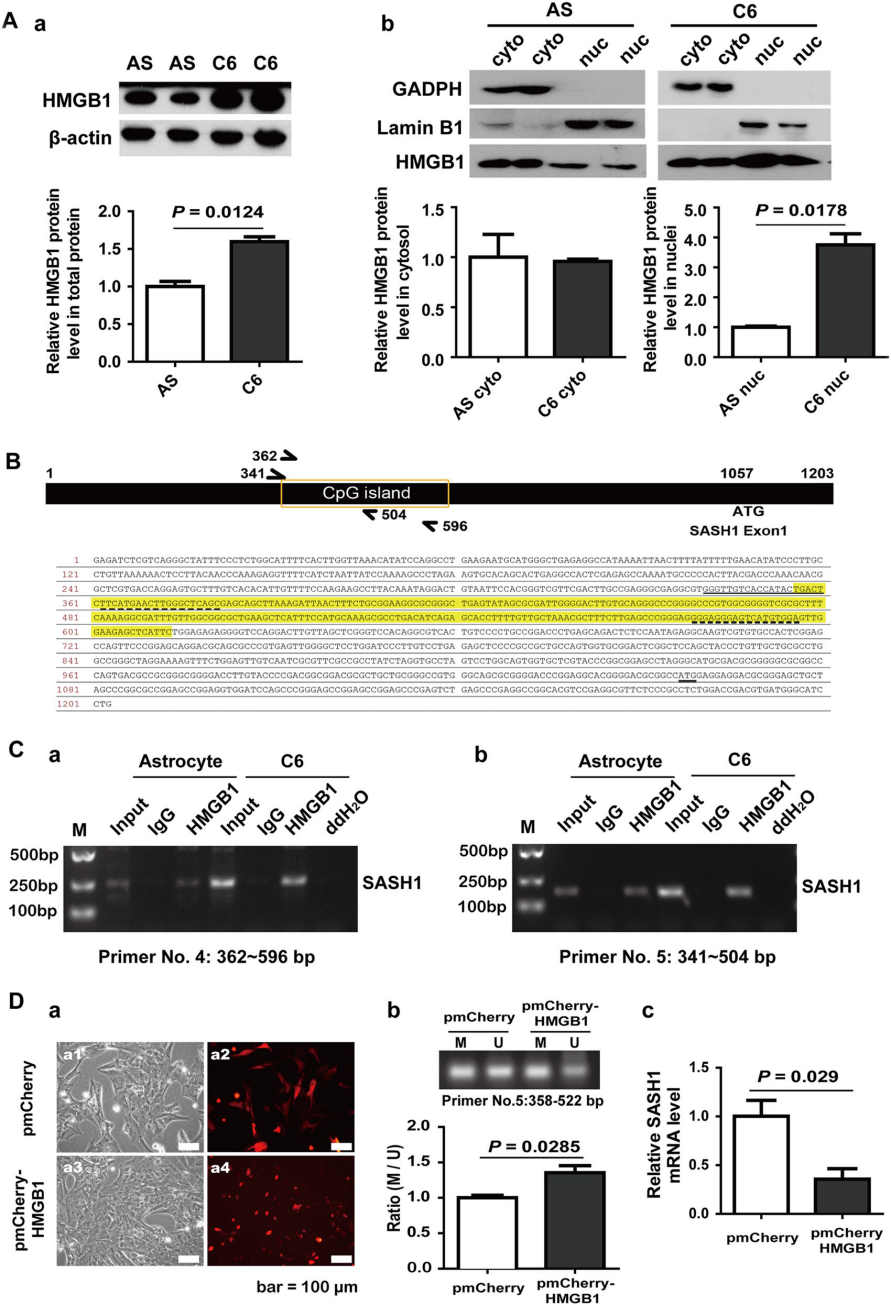
HMGB1 contributes to SASH1 gene methylation. **A** The Western blotting results showed that, a HMGB1 protein was expressed at a high level in C6 cell total protein compared to cultured astrocyte total protein (upper panel, representative blots of duplicate samples; lower panel, statistical results; n = 4, *P* = 0.0124). The results of Western blotting after nuclear and cytoplasmic extract isolation showed that, b HMGB1 protein levels in astrocyte nuclei are significantly lower than those in C6 nuclei. (Upper panel, representative blots of duplicate samples. GAPDH was used as the internal control for cytosolic protein, and LaminB1 was used as the internal control for nuclear protein. Lower panel, the statistical results. *n* = 4, *P* = 0.0178.). **B** Schematic diagram of the CpG sites in the 5′ UTR of the SASH1 gene and the DNA sequences occupied by the PCR primers. **C** Representative results of ChIP analysis. The anti-HMGB1 antibody immunoprecipitated a HMGB1-DNA complex. The DNA region bound by HMGB1 was identified by PCR using SASH1-specific primers (the sequences of primers No. 4 and No. 5 are listed in Table 1), the sites of which are shown in **B**. The input lanes show the PCR products obtained directly from gDNA of astrocytes and C6 cells, the IgG lanes show mouse IgG-immunoprecipitated controls, and the ddH_2_O lanes show the blank PCR controls. **D** HMGB1 overexpression increased the methylation of SASH1 in astrocytes. a Representative images of astrocytes transfected with mCherry-HMGB1 plasmids under bright-field (a1 and a3) and fluorescence (a2 and a4) inverted microscopes and of HMGB1 protein accumulation in nuclei (a4). b Representative PCR results for the SASH1 promoter region using primers (shown in Table 1) specific for methylated (M) and unmethylated (U) gDNA in astrocytes transfected with mCherry or mCherry-HMGB1 constructs. The bar graph (lower panel) shows the M-to-U product ratios. The data are presented as the mean ± SE, *n* = 3, pmCherry-HMGB1 vs. pmCherry, *P* = 0.0285. c qRT-PCR results showing that HMGB1 overexpression in astrocytes could significantly decrease SASH1 mRNA levels. *P* = 0.029

A previous study reported that HMGB1 could regulate gene transcription through chromatin-specific remodeling^18^. We speculated that HMGB1, a well-known multifunctional factor, may be involved in SASH1 gene methylation. As shown in Fig. 5A, using nuclear and cytoplasmic extract isolation and Western blotting, we found that HMGB1 protein expression was increased in C6 whole-cell lysates compared with astrocyte lysates (Fig. 5A, a) and was especially increased in C6 nuclei (Fig. 5A, b). Therefore, we used a ChIP assay to examine whether HMGB1 binds to the CpG sites of the SASH1 gene. As shown in Fig. 5B, C, the HMGB1 antibody could pull down more DNA fragments of predicted CpG island regions from 362 bp to 596 bp and from 341 to 504 bp in the 5′ UTR of the SASH1 gene derived from C6 cells than in that of the SASH1 gene derived from astrocytes. The results revealed that more HMGB1 protein occupies the CpG sites of the SASH1 gene in C6 glioma cells than in astrocytes and that this increased occupation may contribute to the methylation of these sites in C6 cells.

We further tested whether HMGB1 overexpression could increase the methylation level of the SASH1 gene. As shown in Fig. 5D, a, after transfection of cells with mCherry-HMGB1 plasmids, HMGB1 protein accumulated in astrocyte nuclei. Then, we used the MSP method to examine the methylation level of the SASH1 gene. As shown in Fig. 5D, b, HMGB1 protein overexpression significantly increased the methylation level of the SASH1 gene 5′ UTR region (+358 bp to +522 bp). This DNA fragment is precisely within the HMGB1 antibody recognized region. In addition, the SASH1 mRNA level was found to be significantly decreased (Fig. 5D, c) using qRT-PCR detection. These results indicated that HMGB1 could decrease SASH1 gene transcription by facilitating methylation.

## Discussion

Accumulating evidence indicates that SASH1 may be a tumor suppressor gene and that its expression is either decreased or lost in most cancers, including human glioma. The mechanism by which SASH1 plays a role in normal astrocytes and the processes that lead to the decrease or loss of SASH1 expression are not clear to date. Our previous study found that SASH1 expression was much higher in nontumorous tissues than in glioma tissues and that SASH1 expression levels were closely correlated with glioma grade^9^. SASH1 expression is lower in higher-grade tumors, and vice versa. This result suggested an association between gradually declining SASH1 expression and glioma progression.

The property of invasive growth makes glioma an interesting research target^19^. In 2011, Martini et al. reported that SASH1 interacts with the actin cytoskeleton and stimulates cell-matrix adhesion in epithelial cells^20^. Therefore, we wanted to investigate whether this adhesive characteristic would be lost in astrocytes when SASH1 function was depleted. For this purpose, we developed SASH1 siRNA and an Adv4-SASH1 virus as tools to deplete or replenish SASH1 expression, respectively, in cultured astrocytes or in C6 glioma cells. First, the inhibitory effect of SASH1 expression on cell proliferation was confirmed by EdU assay, and this result was similar to results reported in previous publications^3,8^.

Furthermore, we used RNAseq to investigate alterations in cellular function due to SASH1 depletion in astrocytes. Functional cluster analysis of the differentially expressed genes revealed that SASH1 protein depletion significantly affected adhesion. In 2016, Chen et al. reported that SASH1 overexpression suppressed the FAK pathway in cervical cancer cells^21^. Recently, SASH1 was reported to be an inhibitor of tumor metastasis^22^. Our study reveals that in cultured astrocytes, the SASH1 protein plays a role in cell adhesion. Depletion of the SASH1 protein markedly increased cell invasion.

The extracellular matrix (ECM) is an important component of the mechanism controlling cell behaviors, including cell adhesion^23^. We chose Laminin, a major ECM protein in the brain, to coat the Transwell chambers to test for alterations in cell invasion when SASH1 expression was manipulated. The results showed that SASH1 knockdown in astrocytes could significantly increase cell invasion.

Laminin, produced by primary cultured astrocytes, is regarded to function via integrin receptors^24^. Milner et al. once reported that αvβ5 and αvβ8 integrins expressed in primary astrocytes play roles in adhesion^25^. Upon detection, we found that the expression levels of the β8 integrin protein could respond to alterations in SASH1 expression. Furthermore, we transfected cells with β8 integrin plasmids combined with SASH1 siRNA, and the results showed that β8 integrin upregulation in astrocytes could attenuate the increases in cell invasion due to SASH1 depletion. Therefore, our present study suggested that β8 integrin downregulation, which was induced by SASH1 depletion, was involved in attenuating cell adhesion in cultured astrocytes. However, what causes the downregulation of SASH1 gene expression in glioma is unknown. Previously, Zeller et al. noted that they did not find any mutations in the SASH1 gene CDS region, and they suggested that promoter methylation may contribute to SASH1 downregulation in cancers^1^. Sheyu et al. reported some methylation sites of the SASH1 gene promoter in breast cancer cells^26^. Previous studies have documented SASH1 methylation in smoke-related diseases and in cancers^27,28^. Weidmann et al. reported that SASH1 is strongly associated with smoking-linked atherosclerosis^29,30^. Therefore, we compared the methylation statuses of the SASH1 gene in astrocytes and C6 cells. Our results showed that the SASH1 gene in C6 cells has a high methylation level compared to that in astrocytes. However, the difference from previous reports is that our experiments found that the CpG sites were in the 5′ UTR of the SASH1 gene.

HMGB1, a highly conserved nucleoprotein, has been reported to play a role in gliomagenesis and glioma progression^31^. In this study, we verified that HMGB1 expression is increased in C6 glioma cells compared with rat primary astrocytes. We further demonstrated that C6 cells express high levels of HMGB1 with large amounts in their nuclei. We know that HMGB1, a chromatin-binding protein, can facilitate DNA construction and regulate the DNA transcription of some target genes^18^. We demonstrated HMGB1 binding to the CpG islands of the SASH1 gene by ChIP assay. Finally, we demonstrated that HMGB1 overexpression in astrocytes could increase the methylation level of the SASH1 gene. In summary, our study found that in normal primary astrocytes, the scaffold protein SASH1 could play a role in maintaining cell adhesion on Laminin as an extracellular matrix protein. As SASH1 is a tumor suppressor, SASH1 knockdown could decrease β8 integrin expression, resulting in a decrease in cell adhesion. We also verified in the present study that HMGB1, a protein present in large amounts in glioma cell nuclei, contributed to SASH1 gene methylation, thereby resulting in downregulated SASH1 expression.

## Materials and methods

### Rat primary astrocyte and C6 glioma cell culture

Newborn rat pups (P1) were obtained from the Laboratory Animal Center of Nantong University (Nantong, China). All animal surgeries were conducted in accordance with the institutional animal care guidelines and with the National Institutes of Health (Bethesda, MD) guidelines. The primary astrocytes were prepared as previously described^32,33^; cultured in DMEM (Invitrogen, Grand Island, NY) supplemented with 10% fetal bovine serum (FBS; Invitrogen, Grand Island, NY), 0.5 mM glutamine (Invitrogen, Grand Island, NY), and 1% penicillin–streptomycin (P-S; Invitrogen, Grand Island, NY); and incubated in a humidified atmosphere of 95% air and 5% CO_2_ at 37 °C. Briefly, cerebral cortex tissues from P1 rat pups were isolated aseptically, and the meninges were removed. The tissues were dissected out, digested, and gently dropped through a sterile 75-μm Nitex mesh. The cell suspension was used to seed tissue culture flasks. When the cells were confluent, the flasks were shaken at 150 rpm for 16 h to purify the cultures. The successful purification of the subcultured astrocytes was confirmed by glial fibrillary acidic protein (GFAP, Cat. 12389T, CST) immunocytochemical staining, and astrocyte cultures were considered appropriate for use when they were 95% positive for GFAP, as we specified in a previous study^34^.

C6 cells purchased from the Shanghai Cell Bank of the Chinese Academy of Sciences were cultured in the recommended medium, DMEM containing 10% FBS and 1% P-S, under the supplier’s recommended conditions of 37 °C under a 5% CO_2_ atmosphere.

### SASH1 siRNA and AdV-SASH1 treatment and plasmid transfection

siRNAs were designed and synthesized by Biomics Biotechnologies Inc. (Nantong, China). The sequences of the universal control siRNA and the gene-specific SASH1 siRNA are listed in Table 1. One day prior to the experiment, the cultured astrocytes were seeded into 24-well plates at an initial density of 3 × 10^4^ cells/well in 0.5 ml of culture medium. The cultured astrocytes were transfected using Lipofectamine 2000 Transfection Reagent (Invitrogen, Grand Island, NY) according to the manufacturer’s protocol.

**Table 1:** Oligonucleotides, plasmids and antibodies used in this study

Usage	Target	Sequence (5′–3′) or Cat No.
qRT-PCR	GAPDH sense	ccatcactgccactcagaagact
	GAPDH antisense	acattgggggtaggaacacg
	SASH1 sense	ggtggaactgttgcaggaat
	SASH1 antisense	gttggactccgtggatgact
	β8 integrin sense	tcttgattgggttgctt
	β8 integrin antisense	tttctcgtcggtaggtt
Plasmid	pEGFP-N1-integrin β8	refer to NM_ 001108726.1
	pmCherry-HMGB1	refer to NM_ 012963.2
siRNA	Control sense	uucuccgaacgugucacgutt
	Control antisense	acgugacacguucggagaatt
	SASH1 sense	ccagcaguacgcagauuautt
	SASH1 antisense	auaaucugcguacugcuggtt
Methylation-specific PCR	Methylated forward	ggatttagttcgggagttggagtc
	Methylated reverse	accaaaatacccatcacgtcgat
	Unmethylated forward	tggatttagtttgggagttggagtt
	Unmethylated reverse	tcaccaaaatacccatcacatcaat
Bisulfite sequencing	Forward	tgattttttatgaatttgggtttag
	Reverse	aaataaactcttccaactccacataac
ChIP	Primer No.4 sense	ttcatgaacttgggctcagc
	Primer No.4 antisense	gggagggagtcatgtgga
	Primer No.5 sense	gggttgtcaccatactgact
	Primer No.5 antisense	cgatttgttggcggcgc
For identification-Methylation PCR	FI-methylated forward	gattttttatgaatttgggtttagc
	FI-methylated reverse	cataaaaataaacttcaacgccg
	FI-Unmethylated forward	attttttatgaatttgggtttagtga
	FI-Unmethylated Reverse	taaaaataaacttcaacaccacc
Antibodies	SASH1	bs-6099R, Bioss Co.
	β-actin	14755-1-AP, Proteintech Co.
	β8 integrin	ab80637, Abcam Co.
	GFAP	12389T, CST Co.
	GAPDH	60004-1-Ig, Proteintech co.
	Lamin B1	13435s, CST Co.
	HMGB1	H9539, Sigma Co.

Adv4-NC and Adv4-SASH1 were generated and purified by GenePharma Suzhou. For in vitro stimulation, the cultured C6 glioma cells were seeded into 24-well plates one day before the experiment. The cells were infected with Adv4-NC and Adv4-SASH1 at a multiplicity of infection (MOI) of 800 at 37 °C for 4–6 h.

The plasmids pEGFP-N1-Integrin β8 and pmCherry-HMGB1 were synthesized by General Biosystems (Anhui, China) Co. Ltd. Before transfection, cultured astrocytes were seeded into 6-well plates at an initial density of 2 × 10^6^ cells/well in 2 ml of culture medium. The cultured astrocytes were transfected using a Nepa Gene electroporator (NEPA21) according to the manufacturer’s protocol, and the plasmids were used at a density of 10 μg/well.

### qRT-PCR, Western blotting and RNAseq analysis

Total RNA was extracted from astrocytes and C6 glioma cells with TRIzol (Gibco, CA, USA). cDNA was synthesized with an Omniscript RT Kit (Qiagen, Dusseldorf, Germany), and real-time PCR was performed using a DyNAmo Flash SYBR Green qPCR Kit (Thermo Fisher Scientific, MA, USA) following the supplier’s instructions. The primers used to detect SASH1, integrin β8, and the internal control gene GAPDH are listed in Table 1. Western blotting was performed according to standard protocols using the following antibodies: anti-SASH1 antibody (1:500, bs-6099R, Bioss), anti-Integrin β8 antibody (1:1000, ab80637, Abcam), and donkey anti-mouse or anti-rabbit IRDye (1:10,000, Rockland, Limerick, PA, USA). The immunoblots were analyzed using the Odyssey densitometry program (LI-COR, Lincoln, NE, USA). After the astrocytes were transfected with control siRNA or SASH1 siRNA for 3 days, all cells were collected in TRIzol and sent to the gene analysis center at the company 1Gene (http://www.1gene.com.cn).

### Cell proliferation and invasion assays

As described in our previous study^34^, we evaluated cell proliferation using a Cell-Light™ EdU DNA Cell Proliferation Kit (RiboBio, Guangzhou, China). Briefly, the cells were resuspended and seeded at a density of 1 × 10^5^ cells/ml in 96-well plates. At the indicated time after treatment, 50 μM 5-ethynyl-2′-deoxyuridine (EdU) was applied to the cells. After incubation for an additional 24 h, the cells were fixed with 4% formaldehyde in PBS for 30 min. The cells were then assayed, and cell proliferation (the ratio of EdU-positive cells to all cells) was analyzed by using images of randomly selected fields obtained with a DMR fluorescence microscope (Leica Microsystems, Bensheim, Germany).

Cell invasion was examined using 6.5 mm Transwell chambers with 8 μm pores (Corning Lnc., Coring, NY, USA). The upper chamber was coated with Laminin (1:500, Cat. 23017–015, Gibco). A volume of 200 μl of culture medium containing 2 × 10^3^ dissociated cells was seeded in each Laminin-coated chamber, and 600 μl of complete medium was added to each lower chamber. After the cells were cultured for 12–16 h, the cells on the upper chambers were scraped away, whereas the invaded cells on the lower surface were fixed in 4% paraformaldehyde and stained with 0.1% crystal violet before being imaged and counted using a DMR inverted microscope. The assays were performed three times, once using triplicate wells.

### Cell adhesion assay

We used the CCK8 method to evaluate cell adhesion. Briefly, 96-well plates were coated with Laminin or Fibronectin (1:500, Cat. PHE0023, Gibco) overnight at 4 °C. After being transfected for 3 days, the cells were reseeded in 96-well plates and cultured. At the indicated time points, nonadherent cells were removed by washing with PBS, fresh medium with CCK8 reagent was added, and the absorbance was measured with a microplate reader.

### Methylation-specific PCR and bisulfite sequencing

The methylation statuses of the CpG islands in the SASH1 gene promoter region were first investigated by methylation-specific PCR (MSP), as reported previously^17^. If the CpG sites analyzed by MSP are methylated, the methylated (M) band is present. If the sites are unmethylated, the unmethylated (U) band is present. Occasionally, both bands are present if the sites are partially methylated. Genomic DNA extracted from astrocytes and C6 cells was treated with bisulfite reagents (Zymo Research, Irvine, CA). This treatment transforms the unmethylated cytosine into thymine, while the methylated cytosine is unchanged. A total of 20 ng of bisulfite-modified DNA was subjected to PCR amplification and later sequenced using an ABI 3700 automated sequencing system (Applied Biosystems). The MSP primers for the SASH1 gene, which were designed using online software (www.utogene.org/cgi-bin/methprimer), are listed in Table 1. The methylation status was then validated by bisulfite sequencing. The primers used to amplify the predicted CpG-rich region of the SASH1 gene are shown in Table 1.

### Chromosome immunoprecipitation (ChIP)

As described in our previous study^35^, a ChIP assay was performed using an Enzymatic Chromatin IP Kit (CST) according to the manufacturer’s instructions. In brief, astrocytes and C6 cells were fixed in 37% formaldehyde for 10 min at room temperature, and glycine was added to quench the unreacted formaldehyde. The cells were washed with PBS and collected. The cells were sonicated three times for 20 seconds each, a small fraction of sonicated material was retained as the sample, and the remainder was incubated with an antibody against either HMGB1 or IgG. The immunoprecipitated complexes were collected using protein G-agarose beads and washed with elution buffer. The crosslinking of the protein–DNA complexes was reversed at 65 °C for 30 min followed by treatment with 6 μl of 5 M NaCl and 2 μl of proteinase K for 2 h at 65 °C. The DNA was extracted with wash buffer. The pellets were suspended in wash solution and subjected to PCR amplification. We used two pairs of primers (No. 4 and No. 5) to verify the results, and the sequences are listed in Table 1.

### Statistical analysis

Measurements were performed on at least three replicates. The data are expressed as the mean ± SE and were analyzed by one-way analysis of variance and unpaired Student’s *t*-test when necessary. Differences with a *P* < 0.05 were considered statistically significant.

## Supplementary information

Supplementary Figure legend

S1

BiologicalInfoAnalysisReport
