# Supplementary material for: HMGB1 contributes to SASH1 methylation to attenuate astrocyte adhesion
Source: Cell Death Dis. 2019 May 28;10(6):417. doi: 10.1038/s41419-019-1645-7 (PMC6538612; doi:10.1038/s41419-019-1645-7)
Supplement: Supplementary file 1 — Supplementary Figure legend [file 41419_2019_1645_MOESM1_ESM.docx]

**HMGB1 contributes to SASH1 methylation to attenuate astrocyte adhesion**

**Supplementary Figure legend**

**Figure S The results of biological informatics analysis**

The sequencing library was sequenced on a HiSeq platform (Illumina, San Diego, CA, USA). We used the HTSeq statistical package to compare the Read Count values for each gene to the original expression of the gene and then used FPKM values to standardize the expression. Then, we used DESeq to analyze the differentially expressed genes under the following screening conditions: an expression difference multiple |log2FoldChange| > 1 and a significant *P*-value < 0.05. We used the R language Pheatmap software package to perform bidirectional clustering analysis of all the differentially expressed genes. We created a heatmap showing the expression levels of the same gene in different samples and the expression patterns of different genes in the same sample with the Euclidean method to calculate the distance and the complete linkage method to cluster. Next, we mapped all the genes to terms in the Gene Ontology database and calculated the number of differentially expressed genes enriched for each term. Based on the whole genome, the terms with significant enrichment of differentially expressed genes were determined by hypergeometric distribution. The purpose of GO enrichment analysis was to obtain GO functional terms with significant enrichment for the differentially expressed genes, thus revealing the possible functions of the differentially expressed genes in the samples. In addition, we counted the numbers of differentially expressed genes at different levels of KEGG pathways, and we then determined the main metabolic pathways and signaling pathways in which the differentially expressed genes participated.

A, Heatmap and hierarchical clustering analysis results for the 205 differentially expressed genes. The expression values are represented in shades of red and blue, indicating expression above and below the median value, respectively.

B, GO analysis results showing the potential biological processes involving the differentially expressed genes.

C, KEGG analysis results showing the potential signaling pathways involving the differentially expressed genes.
