# Supplementary figures and images for: HMGB1 contributes to SASH1 methylation to attenuate astrocyte adhesion
Source: Cell Death Dis. 2019 May 28;10(6):417. doi: 10.1038/s41419-019-1645-7 (PMC6538612; doi:10.1038/s41419-019-1645-7)

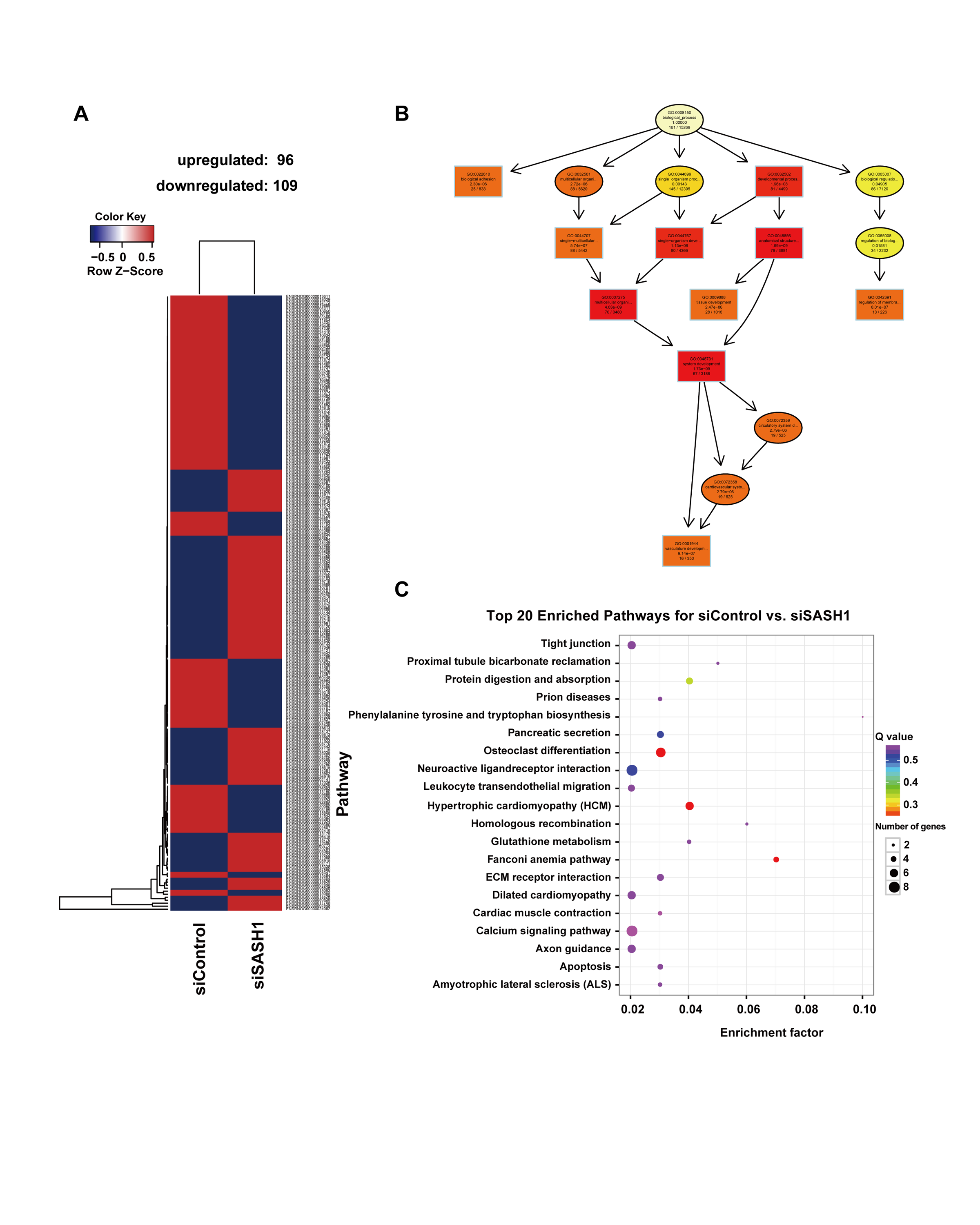

Supplement: Supplementary file 2 — S1 [file 41419_2019_1645_MOESM2_ESM.tif]
